# A unified 3D reconstruction of microscopy and MRI in a brain showing Alzheimer's disease‐related neuropathology

**DOI:** 10.1111/bpa.70039

**Published:** 2025-09-11

**Authors:** Anneke Alkemade, Pierre‐Louis Bazin, Evgeniya Kirilina, Kerrin Pine, Andreas Herrler, Ronald L. A. W. Bleijs, J. Max Kros, Lysanne Groenewegen, Sanne M. M. Vermorgen, Laura E. Jonkman, Dick F. Swaab, Rawien Balesar, Nikolaus Weiskopf, Birte U. Forstmann

**Affiliations:** ^1^ Integrative Model‐Based Neuroscience Research Unit University of Amsterdam Amsterdam The Netherlands; ^2^ Full Brain Picture Analytics Leiden The Netherlands; ^3^ Department of Neurophysics Max Planck Institute for Human Cognitive and Brain Sciences Leipzig Germany; ^4^ Neurocomputation and Neuroimaging Unit, Department of Psychology and Educational Science Free University Berlin Berlin Germany; ^5^ Department of Anatomy and Embryology, Faculty of Health and Life Sciences Maastricht University Maastricht The Netherlands; ^6^ Department of Anatomy, University Medical Center Utrecht Utrecht University Utrecht The Netherlands; ^7^ Department of Pathology Erasmus Medical Center Rotterdam The Netherlands; ^8^ Department of Pathology Amsterdam UMC Amsterdam The Netherlands; ^9^ Netherlands Brain Bank Netherlands Institute for Neuroscience Amsterdam The Netherlands; ^10^ Department of Anatomy and Neurosciences Amsterdam UMC, and Amsterdam Neuroscience, Brain Imaging, and Neurodegeneration Amsterdam The Netherlands; ^11^ Department of Neuropsychiatric Disorders The Netherlands Institute for Neuroscience, An Institute of the Royal Netherlands Academy of Arts and Sciences Amsterdam The Netherlands; ^12^ Felix Bloch Institute for Solid State Physics, Faculty of Physics and Earth Sciences Leipzig University Leipzig Germany; ^13^ Wellcome Centre for Human Neuroimaging, Institute of Neurology University College London London UK

**Keywords:** 3D reconstructions, Alzheimer's disease, post mortem, ultra‐high field MRI, whole brain histology

## Abstract

To bridge between detailed post‐mortem neuropathological assessments and Magnetic Resonance Imaging (MRI), we have created and share a three‐dimensional (3D) account of an entire human brain with an intermediate Alzheimer's disease neuropathologic change. We combined multimodal imaging, using cryosectioning, histology, immunocytochemistry, and quantitative ultra‐high field 7 Tesla (T) magnetic resonance imaging (MRI) at submillimeter resolution. Amyloid‐β and phosphorylated‐tau immunoreactivity, cell soma, and nerve fibers were visualized, together with quantitative MR parameters. All data were coaligned with at 200 μm resolution and are openly shared. The use of whole‐brain sections allows for a detailed assessment of neuropathological alterations, revealing clear differences between the left and right hemispheres in terms of pathological load of amyloid‐β and phosphorylated‐tau in a single brain showing Alzheimer's disease neuropathologic change. This resource opens the door for a combination of detailed correlations between neuroimaging and neuropathological microscopy observations, as well as for detailed MRI validation.

## Graphical abstract

Post mortem MRI was combined with neuropathological assessments at 600 μm intervals throughout the brain. Through this approach, a three dimensional account of an entire human brain with an intermediate Alzheimer's disease neuropathologic change was created. Data were reconstructed, coaligned, and combined in a downloadable resource that allows detailed correlations between neuroimaging data and microscopy observations.

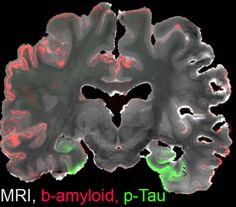

## 1 INTRODUCTION

Alzheimer's disease (AD) is the number one neurodegenerative disease affecting the population worldwide. Non‐invasive imaging techniques allow the uncovering of neuropathological alterations already during life; however, a definitive neuropathological diagnosis can only be made post mortem through the evaluation of the accumulation of extracellular amyloid‐β depositions forming plaques and the intracellular accumulation of hyperphosphorylated tau forming neurofibrillary tangles [1].

In many neuropathological assessments, the post mortem histological observations confirm the clinical diagnosis made during life. However, mismatches occur, and it is not uncommon that cognitively intact elderly show clear neuropathological alterations matching an advanced stage of AD [2, 3, 4, 5]. Imaging efforts are being developed to provide reliable non‐invasive biomarkers for in vivo neuropathology [6], but to what extent magnetic resonance imaging (MRI) results reproduce the imaging profiles observed using microscopy for assessment of the pathology requires further investigation. Interestingly, in vivo imaging approaches have the potential to provide the visualization of whole‐brain neuropathological profiles, but the same does not hold for routine post mortem neuropathological investigations. Post mortem microscopy assessments commonly rely on a select number of tissue blocks used to perform AD neuropathologic staging [1, 7, 8, 9]. Using such a limited sampling procedure comes with the risk of missing key information on regions that are not included, but a whole‐brain 3‐dimensional (3D) reconstruction of the distribution of the amyloid‐β and phosphorylated (p‐)tau protein accumulation using microscopy techniques has not been published so far. With the development of whole brain and tissue block processing and analyses pipelines [10, 11, 12, 13, 14, 15, 16, 17, 18, 19, 20, 21], the required methodology to create full 3D reconstructions of histological profiles in individual human brain donors has become available. Processing pipelines allow systematic MRI scanning, sectioning, and (immuno)histochemical staining, followed by digital reconstruction of large tissue specimens in the same space [10, 11, 12, 13, 14, 16, 19, 20, 21, 22]. Our group has previously published combined MRI and microscopy maps in two non‐demented controls, and a total of four non‐demented whole human brains based on full histological coverage have been reported to date [13, 23]. The resulting unified maps allow the biophysical modeling of quantitative (q) MRI parameters and the linking of MRI to the underlying anatomical characteristics [24]. To achieve similar goals for the imaging of AD‐related neuropathologic change, qMRI can be combined with immunolabeling of p‐tau and amyloid‐β pathology, Bielschowsky staining, and Nissl staining. We therefore set out to extend our previous efforts through the processing of tissue of a donor with AD‐related neuropathological alterations. Through careful selection of the antibodies used in staining procedures and tailoring of the applied MRI protocols, for example, through focusing on iron accumulation which allows detailed mapping of AD‐related neuropathologic change, we provide a full reconstruction of a brain affected by neuropathological alterations [14, 25, 26]. This brain was obtained from a donor registered as a non‐demented control, but who presented with substantial neuropathological alterations during routine assessments performed as part of our histological processing. The resulting descriptive dataset allows relating structural MRI parameters to the deposition of AD proteins including p‐tau and amyloid‐β. In addition to providing proof of concept, the dataset will be of substantial scientific interest, despite the limitation that a single individual brain will not provide insights into interindividual variation, which is important to map given the strong interindividual variability in disease progression and clinical presentations of AD [27].

## 2 METHODS

### 2.1 Case description

A donor head (04‐2017, Female, 91 years, breast carcinoma, pulmonary infection) was obtained from the body donation program of the University of Maastricht following a whole‐body perfusion. Written consent for whole‐body donation was obtained 6 years before death. Clinical data was very limited and did not indicate any comorbidities. The donor was registered as a non‐demented control and did not reside in an assisted living facility.

### 2.2 7 Tesla quantitative MRI and tissue processing

Quantitative MRI maps of relaxation rates (R1, R2*) and proton density were acquired with 400‐μm isotropic resolution at 7 T, as previously described [14, 26]. Magnetic resonance imaging data was acquired using a MAGNETOM 7 T whole‐body system (Siemens Healthineers, Erlangen, Germany), with a circularly polarized radio‐frequency (RF) transmit/32‐channel receiver head array coil (NOVA Medical Inc., Wilmington, MA). The protocol included three 3D multi‐echo GRE scans with T1, proton density (PD‐) and magnetization‐transfer (MT‐) weightings, RF pulse flip angle (FA) = (38, 7, 7) degrees, number of echoes = (8, 8, 6), echo times (TE) equally spaced between 3.4 and 21.6 ms. MT weighting was achieved by a 4 ms long Gaussian RF pulse (3 k HZ off‐resonance, peak amplitude 2.0 μT, BW = 450 Hz) applied once per TR. The sequence was both RF‐ and gradient‐spoiled. With a matrix size of 480 × 640 × 416 (phase × read × partition) the acquisition time per contrast was 1 h 46 min. Four repetitions of the protocol were scanned to obtain sufficient SNR (total acquisition time 7:04 h). In addition to weighted images, maps of the RF magnetic field were acquired for the purpose of B1 + correction [28]. Subsequently, quantitative maps of longitudinal relaxation rate (R1), proton density (PD), magnetization transfer (MT) and effective transversal relaxation time (R2*) were calculated with the hMRI toolbox (http://www.fil.ion.ucl.ac.uk/spm) within the SPM 12 framework (http://hmri.info) and MATLAB. R2* maps were calculated by ordinary least squares fitting to the multi‐contrast data [29].

After brain autopsy, the tissue was sucrose protected and frozen on dry ice in Tissue‐Tek. Whole‐brain blockface images were acquired for all sections during 200 μm serial coronal cutting as described previously [14]. Hematoxylin and eosin (H&E) staining of selected sections was performed for pathological evaluation by two board‐certified neuropathologists. Neuropathological alterations were initially identified through assessment of the following brain areas: the frontal, insular, temporal, and occipital cortex, hippocampus, cingulum, globus pallidus, caudate nucleus, putamen, substantia nigra, red nucleus, locus coeruleus, basalis pontis, dentate nucleus, olivary nucleus, and the cerebellar cortex. Visual assessments revealed the presence of amyloid‐β plaques as well as dilation of the ventricular system and microbleeds. The tissue processing strategy was then tailored accordingly to systematically map neuropathological alterations associated with AD‐related neuropathology. Tissue sections were sampled at 1:3 (600 μm) staining intervals for Bielschowsky silver staining to provide correspondence to the myelin‐sensitive R1 maps for coregistration purposes. Amyloid‐β plaques were stained in free‐floating sections. In short, sections were permeabilized in formic acid (100%, 20 min at room temperature); after rinsing in Tris buffered saline (TBS) and endogenous peroxidase inactivation using 3% H_2_O_2_ for 30 min at RT, sections were incubated in mouse‐anti‐human amyloid‐β (BioLegend cat no. 800701 [previously Covance cat no. SIG‐39220], clone 4G8, lot no. B286227) at 1:20,000 overnight at 4°C (~0.1 mL antibody solution/cm^2^ section surface area). After rinsing, sections were incubated in biotinylated anti‐mouse (1:800, 1 h at RT). Sections were rinsed in TBS and incubated in avidin biotinylated complex (1:800, 1 h at RT), followed by rinsing in TBS and development using diamino‐benzidine (DAB) for 25 min at RT. Sections were rinsed in supermix (SUMI, 0.05 M Tris, 0.15 M NaCl, 0.5% TritonX‐100, and 0.25% gelatin) to prevent tissue clumping, transferred to TBS‐0.1% Triton X‐100, and mounted onto gelatin‐coated slides. After drying, sections were rehydrated through a series of descending graded ethanols and counterstained with thionin. Subsequently, sections were dehydrated in ethanol, cleared in xylene, and finally embedded in Entellan.

For staining of neurofibrillary tangles, we rinsed sections in TBS, inactivated endogenous peroxidase, and incubated in mouse‐anti‐human PHF‐Tau (Thermo Scientific cat no. MN1020, lot no. WH3346162, clone AT8) at 1:2000 in SUMI overnight at 4°C (~0.1 mL Ab solution/cm^2^ section surface area, constant agitation). After washing in TBS and SUMI, sections were incubated in biotinylated anti‐mouse (1:800, 1 h at RT). Sections were rinsed in TBS and SUMI and incubated in avidin biotinylated complex (1:800, 1 h at RT), followed by rinsing in TBS and development using Diamino‐benzidine (DAB) for 30 min at RT. Sections were washed twice in SUMI and transferred to TBS‐0.1% Triton X‐100 to mount sections onto gelatin‐coated slides. After drying and rehydration, sections were stained with thionin, dehydrated, cleared in xylene, and coverslipped using Entellan. Given that both amyloid‐β and were counterstained with thionin, the staining interval for thionin was 2 to 3.

## 3 DIGITIZATION AND RECONSTRUCTIONS

All sections were imaged at 1200 dpi using a flatbed scanner, and images were used for 3D reconstruction of the brain in blockface space, as described previously using non‐linear 2D registration of each section to the corresponding blockface images, to compensate for tissue distortions caused by the tissue processing [13]. The co‐registration technique used a multi‐scale, multi‐step registration approach based on the non‐linear SyN algorithm in ANTs with optimization of mutual information including the gray–white matter transition. The methods and the resulting registration quality have been described previously [13]. Quality checks were performed using the same analysis of the alignment as in our previous work [13], computing the mean distance between boundaries of adjacent sections processed using the same staining protocol. Subsequently, individual sections were selected that included both cortical and subcortical brain areas, which are commonly used for the assessment of neuropathological alterations, to provide a general overview of AD‐related neuropathology of both the left and right hemisphere. QuPath [30] was subsequently used on these sections to determine the neuropathological load. In QuPath, regions of interest were outlined, and images were thresholded (threshold was set at 20,000 arbitrary units since this setting consistently masked the immunoreactivity for both amyloid‐β and p‐tau), and the percentage of surface covered by immunoreactivity within a region of interest was determined.

## 4 RESULTS

Before histological processing, the donor head was subjected to extensive quantitative 7 T MRI scanning (R1‐maps, R2*‐maps, Quantitative Susceptibility Mapping and Proton Density Mapping) [14]. H&E stainings were performed and assessed under the microscope, revealing an intermediate degree of AD neuropathologic change. This observation triggered us to perform a more detailed evaluation of the amyloid‐β immunoreactivity in selected areas, which confirmed a phase of 3/5 [9], Braak neurofibrillary tangles (NFT) stage of 4/6 [7], together with the presence of neuritic plaques. This resulted in an AD neuropathologic change: A2, B2, C2 according to Montine et al. [1]. Observed amyloid‐beta plaques were more diffuse than classic‐cored. Thorn‐shaped age‐related tauopathy of the astroglia (ARTAG) was present mesiotemporally at the level of the amygdala, as well as in the subpial region. A small number of fuzzy astrocytes were observed in the amygdala. Neuropathological assessments further revealed cerebral amyloid angiopathy (CAA) stage of 1 [31], and agrophylic grain disease (AGD) stage 4/4 [32]. An old infarction was present in the parastriate region of the left occipital lobe, and in the substantia nigra, a small number of inclusions were visible that were not further defined. Subsequent measurements of the area percentage covered by immunoreactivity of the neuropathological load were performed on the digitalized stainings. This data is summarized in Table 1 together with qMRI intensity values.

**TABLE 1:** The qMRI intensity values and corresponding neuropathological loads.

	MTsat (p.u.)		PD (p.u.)		R1 (1/s)		R2* (1/s)		Amyloid‐β (%*1000)		p‐tau (%*1000)		Bielschowsky (a.u.)	
	L	R	L	R	L	R	L	R	L	R	L	R	L	R
Frontal cortex (overall)	0.73	0.74	74.78	73.94	0.95	0.99	32.23	26.66	11.1	8.09	0.91	0.69	443.11	432.38
Superior frontal cortex	0.75	0.83	77.54	74.40	0.99	1.08	40.65	32.26	1.78	2.63	0.75	0.71	439.79	440.11
Medial frontal gyrus	0.70	0.73	74.95	73.72	0.90	0.96	22.42	25.39	6.30	4.33	0.25	0.36	429.98	436.22
Anterior dorsal cingulate cortex	0.84	0.76	76.44	80.41	1.18	1.17	32.10	48.16	0.76	2.82	12.45	20.31	447.61	431.07
Anterior ventral cingulate cortex	0.80	0.77	81.31	85.00	1.16	1.12	43.77	46.48	0.98	1.23	12.07	109.69	423.26	417.23
Posterior dorsal cingulate cortex	0.85	0.91	74.84	74.54	1.18	1.27	26.12	27.51	0.85	0.98	0.54	0.21	435.61	431.92
Posterior ventral cingulate cortex	0.78	0.77	79.74	77.03	1.28	1.33	52.83	65.99	0.75	0.56	3.24	8.95	452.02	450.94
Inferior parietal cortex	0.83	0.63	78.02	73.77	1.07	0.84	63.93	23.73	6.33	32.23	0.26	0.70	442.89	430.37
Superior parietal cortex	0.69	0.76	73.48	72.40	0.97	1.07	27.58	29.36	9.54	16.02	0.23	0.22	440.39	438.50
Insular cortex	0.82	0.78	81.24	77.17	1.07	1.14	36.45	26.54	0.70	1.41	6.79	0.15	442.14	439.54
Medial temporal gyrus	0.79	0.74	72.23	75.74	1.35	0.98	42.19	24.16	2.31	5.56	3.11	1.27	439.99	418.76
Primary visual cortex	0.84	0.86	73.95	76.44	1.19	1.23	32.02	32.51	1.04	1.08	0.23	0.12	456.91	446.60
Amygdala	0.86	0.86	71.29	72.51	1.22	1.25	27.09	28.15	0.27	0.27	35.71	12.92	431.25	454.95
Entorhinal cortex	0.76	0.90	79.85	76.32	1.02	1.26	38.38	28.51	0.51	0.88	85.71	14.80	443.19	441.69
Hippocampus	0.61	0.80	84.02	75.90	0.93	1.18	44.80	26.72	0.24	0.82	62.87	14.81	460.59	422.24
Thalamus	1.17	1.11	76.88	75.85	1.29	1.17	40.75	33.58	0.11	0.18	0.03	0.09	453.52	462.39
Globus pallidus	1.17	1.22	73.57	73.27	1.44	1.46	64.62	59.43	0.02	0.03	0.01	0.01	460.10	480.97
Nucleus accumbens	0.66	0.81	71.60	74.48	0.96	1.37	41.24	37.37	0.28	0.14	1.20	5.20	442.49	446.90
Caudate nucleus	0.72	0.80	79.14	75.57	1.21	1.29	45.38	37.61	0.24	0.96	0.10	0.24	437.70	442.12
Putamen	0.97	0.96	75.70	77.22	1.43	1.43	64.61	65.14	0.01	0.04	0.01	0.06	430.24	436.79
Substantia nigra	1.14	1.11	73.46	76.48	1.42	1.35	66.75	63.02	0.04	0.05	0.15	0.23	414.35	418.41
Red nucleus	1.56	1.54	69.12	70.59	1.60	1.54	64.16	65.90	0.00	0.01	0.01	0.01	502.86	502.83
Locus coeruleus	0.80	0.97	80.03	77.46	1.17	1.15	43.33	35.64	0.03	1.52	1.23	6.08	405.10	376.65
Olivary nucleus	1.65	1.64	70.21	65.12	1.68	1.74	36.58	37.04	2.75	1.16	0.39	0.11	476.55	468.74
Cerebellar cortex	0.98	1.08	75.26	77.74	1.30	1.38	23.93	25.42	0.98	0.72	0.28	0.28	409.16	417.59
Dentate nucleus	1.40	1.61	75.37	71.45	1.51	1.66	60.67	61.23	2.03	3.23	1.00	2.25	482.72	471.80

*Note*: Amyloid‐β, p‐tau present % of the surface area covered by staining.

Abbreviations: a.u., arbitrary units; Mtsat, magnetization transfer saturation, PD, proton density values.

**FIGURE 1:**
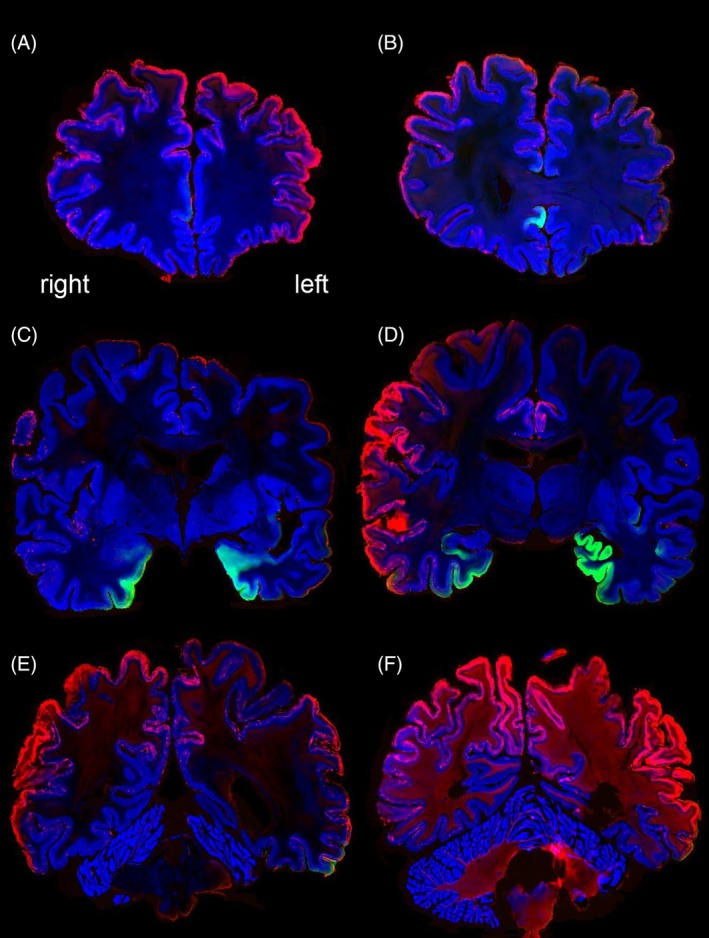
(A–F) showing coronal sections at different rostro‐caudal positions. Blue = Nissl, Red = amyloid‐β, Green = hyperphosphorylated tau. Note the asymmetric distribution of tau pathology in the subcallosal cingulate gyrus (B), as well the asymmetric distribution of amyloid‐β in the cortex.

**FIGURE 2:**
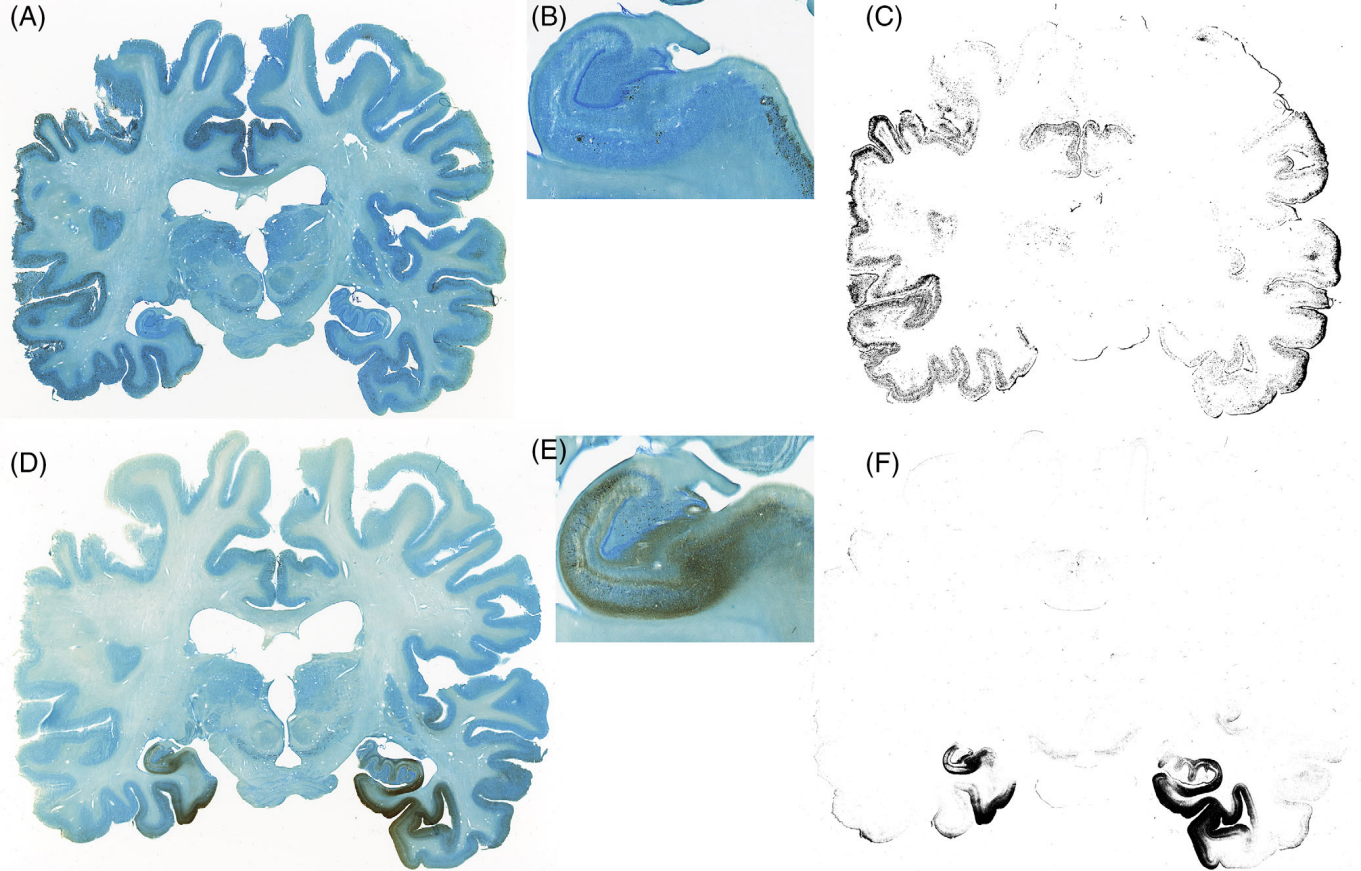
Illustrations of the bright field images of amyloid‐β (A, B), and the deconvoluted image (C) showing the area covered by amyloid‐β, and illustrations of tau pathology staining (D, E) and the area covered (F).

**FIGURE 3:**
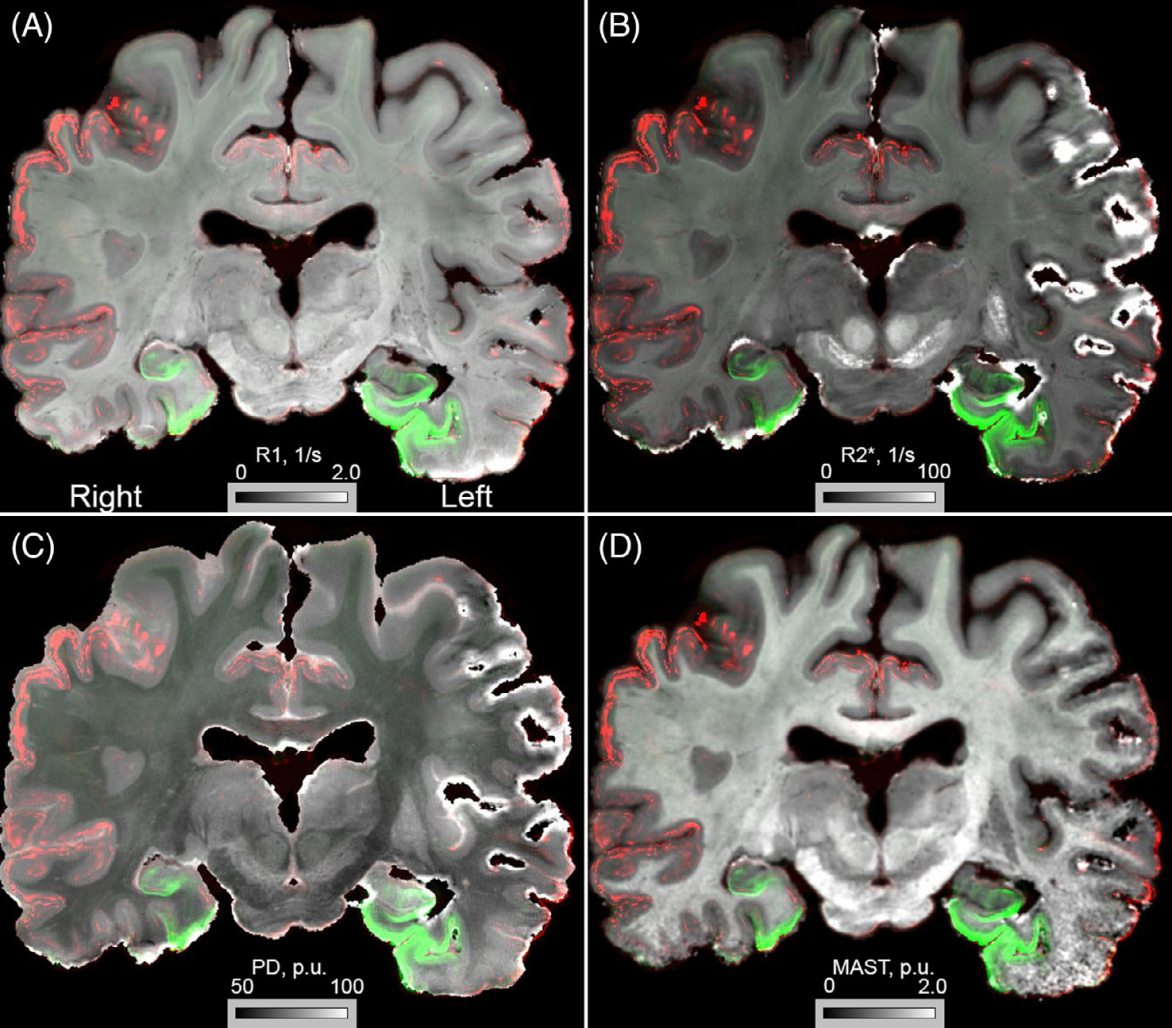
Illustrations of coregistrations of MRI with amyloid‐β (red) and tau pathology (green). (A) R1‐map, (B) R2*‐map, (C) Proton density map, (D) Magnetization Transfer saturation map (MAST).

**FIGURE 4:**
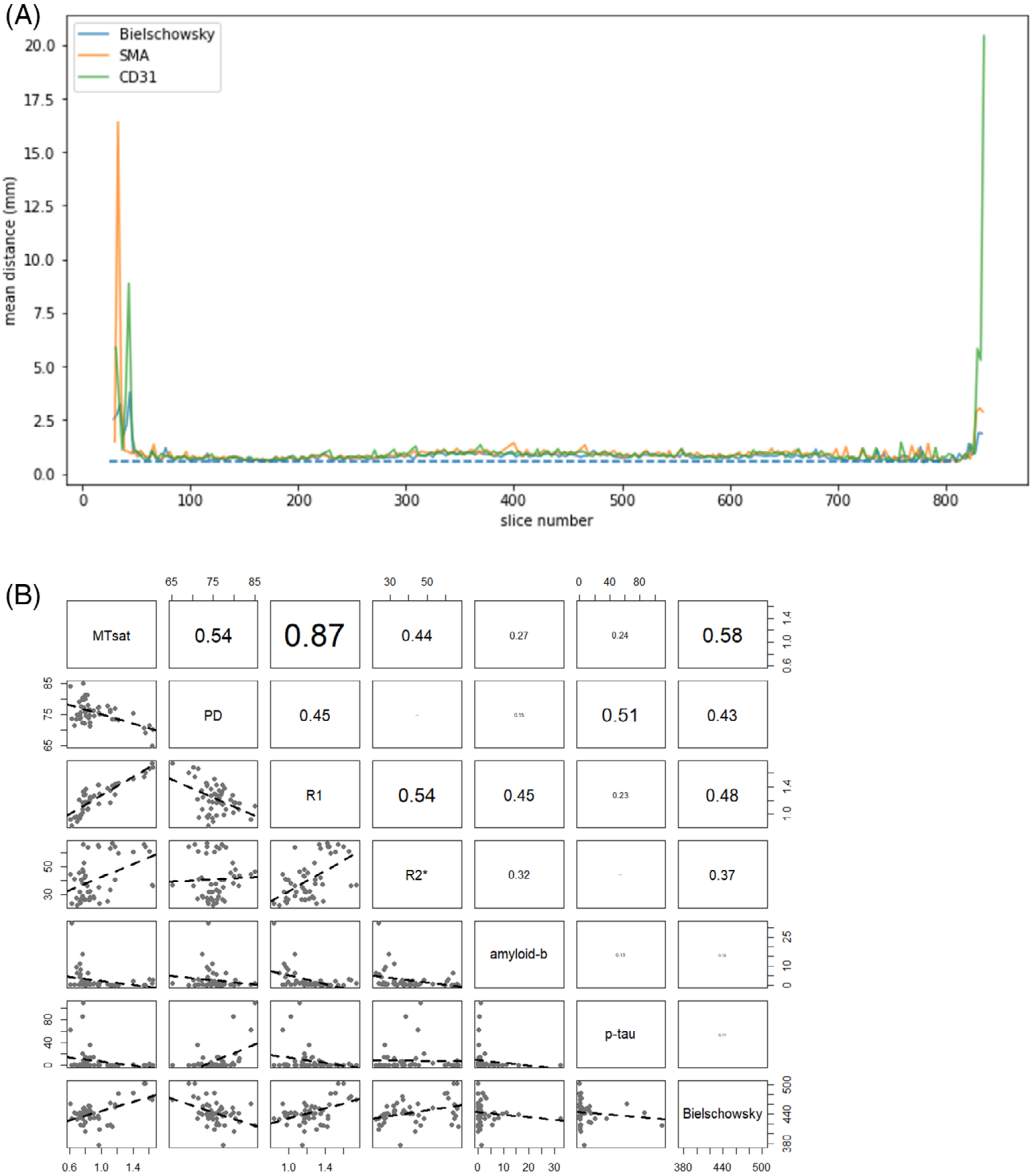
(A) Mean section boundary distance for the three staining contrasts after co‐registration. The dotted line indicates the average section distance of 0.6 mm. (B) Linear correlation coefficients between MRI and histological quantifications. Note that we did not calculate the best‐fitted line through the observations (which may be non‐linear). Additionally, the absence of a linear correlation does not indicate the absence of a complex relationship.

**FIGURE 5:**
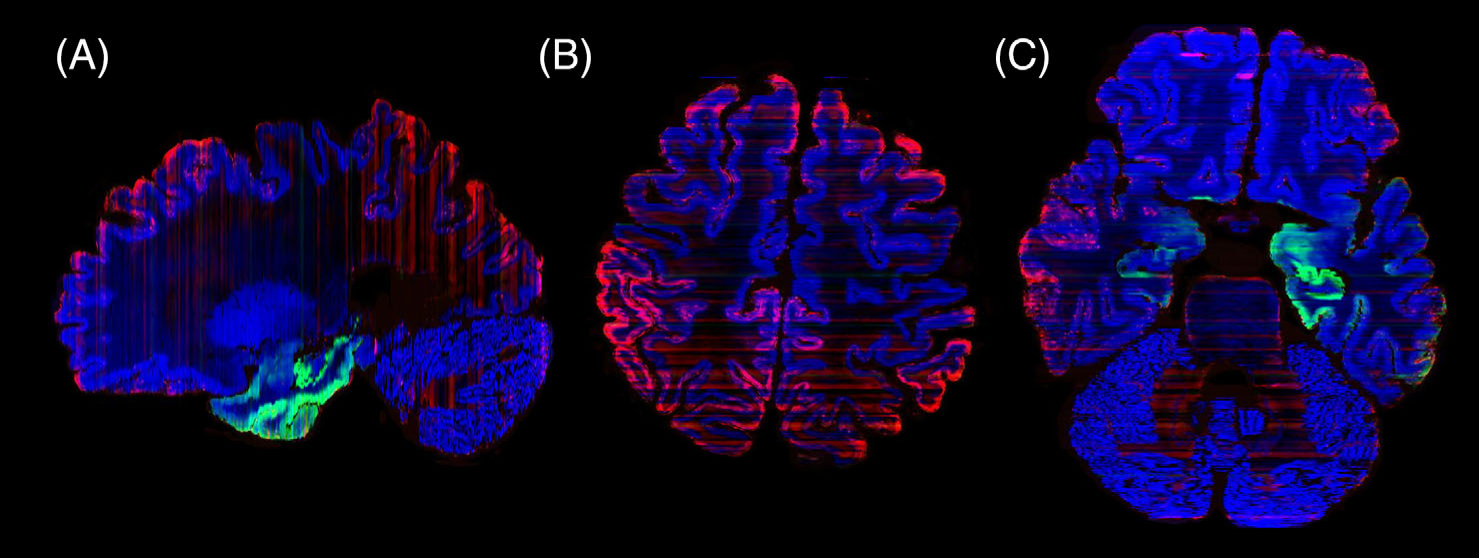
Illustrations of the reconstructions in the (A) sagittal and (B/C) axial plane. Red stripes are caused by differences in background staining intensity resulting from interassay variation in the staining process.

All microscopy slides were subsequently reconstructed into a common space with the 7 T quantitative multiparameter MRI at 200 μm resolution. To allow a more extensive comparison between immunoreactivity and qMRI data, we have shared all registrations of the imaging modalities in blockface space. The reconstruction of the 3D neuropathological alterations at a 200 μm isotropic resolution revealed a broad distribution of amyloid‐β pathology in the neocortex. Amyloid‐β showed a clearly distinct distribution pattern differentially affecting particular cortical areas (Figure 1). Most intense staining for p‐tau pathology was observed in the entorhinal cortex and hippocampus. Strikingly, the right subcallosal cingulate gyrus showed clear p‐tau accumulation, which was not observed in the contralateral hemisphere (Figure 1B). Illustrations of the staining results are presented in Figures 1 and 2. Correspondence between qMRI and histological stainings is shown in Figure 3. Additionally, the mean distance between consecutive section boundaries was 0.85, 0.97, and 1.04 mm for Bielschowsky, SMA, and CD31 stainings, respectively. Of note: larger distances were confined to the most frontal and occipital brain sections (Figure 4A). Linear correlation coefficients were calculated between MRI and histological measures (Figure 4B). A full account of the distribution of the individual staining patterns is illustrated in Video 1 using color RGB labeling of the different immunoreactivity profiles. The alignment of MRI contrasts and amyloid‐β and p‐tau pathology is further illustrated in Video 2A–D, and the quality of the co‐alignments can be appreciated in Figure 5, which shows the reconstructed sagittal and axial planes. The 3D reconstructions were created combining the quantitative MRI with the histological results, which allows direct comparisons of the histological profile and the MRI. Videos can be accessed through the datasharing (DOI: https://doi.org/10.21942/uva.c.7260322.v2).

## 5 DISCUSSION

Here we present a full reconstruction at a 200 μm isotropic resolution of a human brain with intermediate AD‐related neuropathologic change resulting in an A2, B2, and C2 score. We report density measures in Table 1, which should be interpreted with caution since they only represent the results based on a single case. These results provide proof of concept that whole‐brain mapping of disease‐specific proteins can be achieved by adapting a protocol established from specimens of non‐demented controls [13].

We would like to note that to achieve full reconstructions, microscopy slides were digitized with an in‐plane resolution of 21 μm. These data were subsequently down‐sampled to 200 μm. Neuropathological assessment was performed at the highest resolutions, directly on the slides using light microscopy, thereby allowing a more accurate assessment of neuropathological load than possible based on the reconstructions in Figure 1. The inclusion of Bielshowsky staining and staining procedures for an account of the neuropathological load throughout the entire brain allows for quantitative comparisons across modalities and hemispheres. We performed H&E staining on a relatively small number of brain regions. However, it is known that AD‐related neuropathology extends beyond these brain areas, and AD‐related neuropathologic change affects the entire brain in advanced stages of the disease. The observed more focal distribution of p‐tau and the more widespread amyloid‐β immunoreactivity distribution are both consistent with seminal literature in this field on which neuropathological staging is based [7, 33]. The creation of whole‐brain 3D reconstructions of the AD‐related neuropathologic change in combination with detailed quantitative MRI contrasts now allows the study of the neuropathological alterations and their relation to MRI observations throughout the brain with an unprecedented level of detail.

The donor described in this study was registered as a non‐demented control 6 years prior to death, and apart from age, it is important to note that the available (clinical) information was very limited. It is unknown whether the donor had progressed to the clinical stages of AD after initial registration as a brain donor, or whether the donor still had largely intact cognitive function and was resilient to cognitive decline. The latter would fit with the fact that the donor did not live in a care facility. Given the advanced age of the donor (91 years), as well as literature indicating that cognitive function and AD‐related neuropathological change do not show a one‐to‐one correspondence [2, 3, 4, 5], any assumption on the donor's cognitive functioning at the end stages of life would be speculative. Despite the limited clinical information, the created dataset shows a number of interesting results in addition to being a scientific resource. Particularly, the presence of substantial asymmetry across the hemispheres is of potential relevance (see Figure 1), given that often only one hemisphere is used for neuropathological assessments. The question arises as to what extent asymmetrical pathology contributes to potential differences in clinical phenotypes. Earlier studies have already demonstrated the presence of asymmetric cortical neuropathology in AD, which appeared to be associated with the language dominant hemisphere [34, 35]. Our whole‐brain reconstruction now allows us to investigate asymmetry in deeper brain structures as well. Together with our previously shared datasets, and datasets reported by others, the cumulative release of a growing number of individual reconstructed brains is building toward a digital brain collection that will continue to grow across various neurodegenerative diseases, and eventually will allow statistical comparisons [13, 23].

We chose to design the histological staining procedure to visualize AD‐related neuropathologic change consisting of p‐tau, including pretangles as well as tangles in cell bodies and neuropil threads in neuronal processes, and material in dystrophic nerve cell processes of neuritic plaques [36, 37, 38]. Although the amyloid‐β protein was much more widespread, it showed substantial heterogeneity, with highest expression in the left entorhinal cortex (see Table 1). It is important to note that the qMRI signal reflects a complex signal of accumulated neuropathologic proteins and normal brain tissue, and therefore does not provide a direct correspondence to the tissue pathology. The asymmetric labeling of only the right subcallosal cingulate gyrus with p‐tau is in line with previous positron emission tomography (PET) studies reporting an increased tau deposition in the right subcallosal cingulate gyrus in people showing cognitive impairments in AD [39]. The focal distribution of tauopathy in the medial temporal and adjacent cortices observed in preclinical AD is also in line with this work.

Translations between gold standard neuropathological assessment of AD and clinical assessments such as those reported in valuable in vivo databases including the Alzheimer's Disease Neuroimaging Initiative (ADNI) have the potential to contribute to a deeper understanding of in vivo neuropathological alterations as imaged using noninvasive approaches such as MRI (ADNI | Alzheimer's Disease Neuroimaging Initiative [24, 40]). Important work coregistering MRI and microscopy observations in AD has been published by Ushizima et al. [22]. The linking is of particular interest given the importance of a one‐to‐one spatial mapping of clinical alterations associated with AD with the neuropathological alterations observed in histological specimens. The combination of postmortem quantitative MRI with histological processing and full 3D reconstructions allows the creation and sharing of scientific resources for studies on human brain anatomy, creation of detailed brain atlases of small deep brain structures that cannot be readily visualized using MRI as well as MRI validation, for example, through the mapping of image distortions [25]. With the release of this reconstruction of an entire brain from a donor with AD‐related neuropathological alterations including quantitative MRI and immunoreactivity as a resource, we are starting to build an important whole‐brain collection, which we offer without restrictions to researchers worldwide to connect microanatomy, neuroimaging, and systems neuroscience both in the normal and neurodegenerative brain.

## AUTHOR CONTRIBUTIONS

Anneke Alkemade: Conceptualization and execution of the research, data curation, writing of the manuscript. Pierre‐Louis Bazin: Reconstructions and co‐registrations, conceptualization of the research, data curation, editing of the manuscript. Evgeniya Kirilina, Kerrin Pine, and Nikolaus Weiskopf: Quantitative MRI, editing of the manuscript. Andreas Herrler: Tissue selection and processing, editing of the manuscript. Ronald L. A. W. Bleijs: Tissue processing, editing of the manuscript. J. Max Kros and Sanne M. M. Vermorgen: Neuropathological diagnosing, editing of the manuscript. Lysanne Groenewegen and Rawien Balesar: Tissue processing, editing of the manuscript. Laura E. Jonkman: Neuropathological assessments, editing of the manuscript. Dick F. Swaab: Editing of the manuscript. Birte U. Forstmann: Conceptualization, editing of the manuscript.

## FUNDING INFORMATION

This research is financially supported by STW/NWO (BUF, AA), NWO VICI (BUF), the European Research Council (BUF ERC CoG‐Deep Brain [no. 864750] and ERC PoC‐DeepBrainVasc and NW [no. 616905]). ZonMW Open competition (AA, LEJ, BUF). JPND/ZonMW (Grant 73305113, AA). NW received funding from the Deutsche Forschungsgemeinschaft (DFG, German Research Foundation) – project no. 347592254 (WE 5046/4‐2), the Federal Ministry of Education and Research (BMBF) under support code 01ED2210, the BMBF (01EW1711A & B) in the framework of ERA‐NET NEURON.

## CONFLICT OF INTEREST STATEMENT

The Max Planck Institute for Human Cognitive and Brain Sciences and Wellcome Centre for Human Neuroimaging (authors EK, KP, NW) have institutional research agreements with Siemens Healthcare. NW holds a patent on the acquisition of MRI data during spoiler gradients (US 10401453 B2). NW was a speaker at an event organized by Siemens Healthcare and was reimbursed for the travel expenses. AA, P‐LB, AH, RLAWB, JMK, LG, SMMV, LEJ, DFS, RB, and BUF have nothing to disclose.

## ETHICS STATEMENT

Written consent was obtained from the brain donor 6 years prior to death. All procedures were in accordance with the Dutch Burial and Cremation Act.

## Data Availability

All videos are accessible via the following DOI: https://doi.org/10.21942/uva.c.7260322.v2 (Video 1. Bielschowsky (blue), amyloid‐β (red), tau (green). Video 2. A R1 gray, amyloid‐β (red), tau (green). Video 2B. R2* gray, amyloid‐β (red), tau (green). Video 2C. PD gray, amyloid‐β (red), tau (green). Video 2D. MT gray, amyloid‐β (red), tau (green)). Data processing scripts, individual tiffs of all sections, 3D reconstructions in blockface space and registered to MNI‐2009B space, as well as anatomical parcellations and the .csv file of Table 1 are all shared freely and without any restrictions with the scientific community (https://doi.org/10.21942/uva.c.7260322.v2).
